# How Does Aging Affect Social Attention? A Test of Competing Theories Using Multilevel Meta-Analysis

**DOI:** 10.1093/geronb/gbac052

**Published:** 2022-03-12

**Authors:** Kate T McKay, Louisa A Talipski, Sarah A Grainger, Manikya Alister, Julie D Henry

**Affiliations:** School of Psychology, The University of Queensland, St Lucia, Queensland, Australia; Research School of Psychology, The Australian National University, Canberra, Australian Capital Territory, Australia; School of Psychology, The University of Queensland, St Lucia, Queensland, Australia; School of Psychological Sciences, The University of Melbourne, Melbourne, Victoria, Australia; School of Psychology, The University of Queensland, St Lucia, Queensland, Australia

**Keywords:** Gaze, cueing, Social, cognitive aging, Visual attention

## Abstract

**Objectives:**

The present study provides a meta-analytic assessment of how gaze-cued attention—a core social-cognitive process—is influenced by normal adult aging.

**Methods:**

A multilevel meta-analysis of standardized mean changes was conducted on gaze-cueing effects. Age effects were quantified as standardized mean differences in gaze-cueing effect sizes between young and older adult samples.

**Results:**

We identified 82 gaze-cueing effects (*k* = 26, *N* = 919 participants). Of these, 37 were associated with young adults (*k* = 12, *n* = 438) and 45 with older adults (*k* = 14, *n* = 481). Relative to younger adults, older adults had a reduced gaze-cueing effect overall, *g* = −0.59, with this age effect greater when the cues were predictive, *g* = −3.24, rather than nonpredictive, *g* = −0.78.

**Discussion:**

These results provide the clearest evidence to date that adult aging is associated with a reduction in gaze-cued attention. The results also speak to potential mechanisms of this age effect. In line with cognitive decline models of aging, it was demonstrated that when gaze cues were predictive, only younger adults seem to benefit, suggesting that older adults exhibit a particularly reduced capacity to use gaze cues volitionally.

Strong positive associations between high-quality social relationships and well-being are now well established (Caccioppo & Hawkley, 2009). In older adult cohorts, poor social connection is associated with important health-related outcomes including increased depressive symptoms (Chen et al., 2021); a reduced ability to complete activities of daily living (Teo et al., 2017); accelerated cognitive decline (Béland et al., 2005); increased risk of neurodegenerative disease (Livingston et al., 2020); and even premature mortality (Lee et al., 2020). It is therefore important to understand whether the social-cognitive mechanisms that underpin the development and maintenance of strong social relationships are affected by aging. Additionally, when decline is present, it is important to understand the underlying mechanisms responsible for that decline.

Gaze-cued attention, that is, the reallocation of our own attentional resources to those locations toward which others look, is widely regarded as one of the core components of social cognition (Argyle & Cook, 1976; Emery, 2000; Frith, 2008). For instance, a central tenet of the shared-attention model of social cognition (see Stephenson et al., 2021) is that, via early-emerging gaze-detection and gaze-following mechanisms, human beings engage in shared attention with others, and in doing so, observe what they are observing. Through an innate understanding that others *see* what they look at, we infer the content of their minds. In this way, gaze-cued attention is considered to enable and support basic mindreading, from which the more complex Theory of Mind system is thought to emerge (Emery, 2000).

Given this presumed importance for social-cognitive function, an extensive empirical literature trying to index gaze-cued attention has emerged, most of which has relied on the gaze-cueing paradigm (Friesen & Kingstone, 1998). This involves presenting observers with a central face with eyes gazing either left or right followed by a target to be reacted to at either the left or right periphery. There are therefore gaze-cued trials—in which targets are presented at the side toward which the gaze cue is directed—and gaze-miscued trials—in which the target is presented at the side opposite to which the gaze cue is directed. Both trial types are exemplified in Figure 1 (Slessor et al., 2016). Healthy adults consistently demonstrate gaze-cued attention, referred to as the gaze-cueing effect, wherein they respond faster to targets that are cued rather than miscued by the preceding gaze cue (see McKay et al., 2021). This robust gaze-cueing effect has been widely interpreted as evidence that gaze cues are indeed important social perceptual cues in that they clearly inform our behavior during interactions with others (i.e., in line with the central assumptions of the shared-attention model; Stephenson et al., 2021).

**Figure 1.**
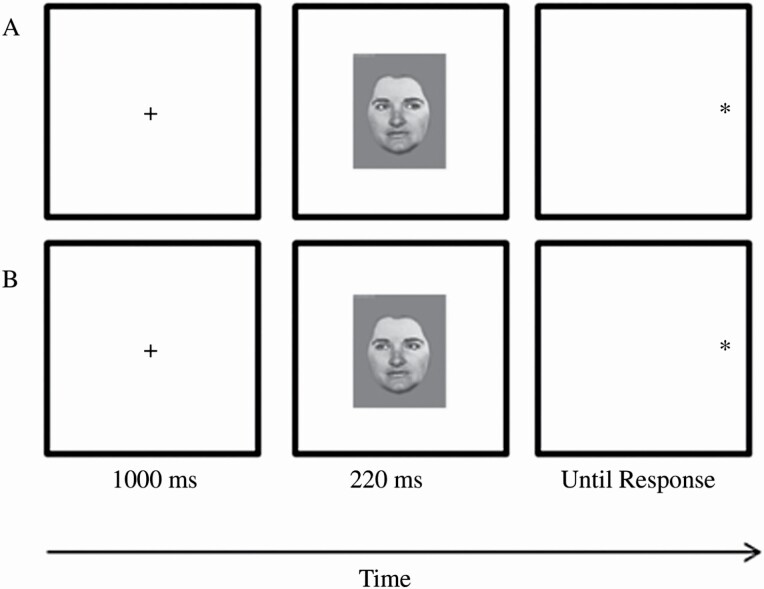
Example gaze-cueing trials from Slessor et al. (2016) showing a gaze-cued trial (Panel A) and a gaze-miscued trial (Panel B).

Importantly, it has been noted that gaze-cued attention can be both reflexive and volitional. That is, in some situations, attentional resources may be allocated via others’ eye gaze in a reflexive manner, while at other times, a more deliberate, volitional type of gaze-cued orienting may be required. To index these different types of orienting, gaze-cueing tasks manipulate cue predictiveness. Predictive gaze-cueing tasks are ones where targets are gaze-cued more often than they are gaze-miscued. Nonpredictive gaze-cueing tasks are ones where targets are gaze-cued exactly as often as they are gaze-miscued. Slessor et al. (2016; Figure 1) asked participants to complete both predictive and nonpredictive gaze-cueing tasks. In the predictive task, two thirds of trials were gaze-cued and one third were gaze-miscued. In the nonpredictive task, there were an equal number of gaze-cued and gaze-miscued trials. Predictive tasks are generally considered to tap into volitional attentional orienting while nonpredictive tasks are generally considered to tap into reflexive attentional orienting (Olk & Kingstone, 2015; see Erel & Levy, 2016 for a review that discusses the relationship between cue predictiveness and orienting mechanisms).

At present, our understanding of how age might influence gaze-cued attention overall, as well as for these specific types of orienting, is unclear. Although the first aging study to address this question identified age-related decline (Slessor et al., 2008), findings from subsequent studies have been mixed, with some also identifying age-related decline (e.g., Bailey et al., 2014; Slessor et al., 2010), but others no age effects (e.g., Deroche et al., 2016; Gayzur et al., 2014). Competing theoretical models—namely, visual attention accounts and cognitive decline models—also generate opposite predictions about how gaze-cued attention might be affected by normal adult aging.

## The Visual Attention Account

While free-viewing images of faces, older adults reliably look more at the mouth and less at the eyes than younger adults (Grainger & Henry, 2020). It has previously been theorized that this visual attention bias contributes to other age-related differences such as older adults’ poorer facial affect recognition (Wong et al., 2005). If this is true, this age-related visual attention bias should particularly affect those social-cognitive behaviors that rely on early reflexive visual attention to eye gaze. Therefore, the visual attention account predicts that age-related decline in gaze-cueing should be greatest when the gaze-cueing task is nonpredictive rather than predictive.

## The Cognitive Decline Model

Although healthy aging is associated with decline in many aspects of cognitive function (Albinet et al., 2012), cognitive operations that rely on automatic or reflexive processes seem to be relatively spared compared to those that rely on more controlled or volitional processes (i.e., in line with classical dual process theories; Craik & Jacoby, 1996; Hasher & Zacks, 1988). The cognitive decline model therefore predicts that age-related decline in gaze-cueing should be greatest when the gaze-cueing task is predictive rather than nonpredictive, directly contrary to the visual attention account.

## The Present Study

We aimed to quantify the magnitude and direction of gaze-cueing age effects overall as well as separately for predictive and nonpredictive gaze-cueing tasks to provide a test of the two competing theories.

## Method

The study design was preregistered at the Open Science Framework on 5 May, 2021 (http://osf.io/w4ktz) prior to data collection (i.e., before database searches). Predictions and analysis plans were added 23 October, 2021 (http://osf.io/j7p2e). At that time, data extraction had not begun (Author Note 1). The Data files and annotated R scripts are provided within the latter.

### Study Selection

**Figure 2.**
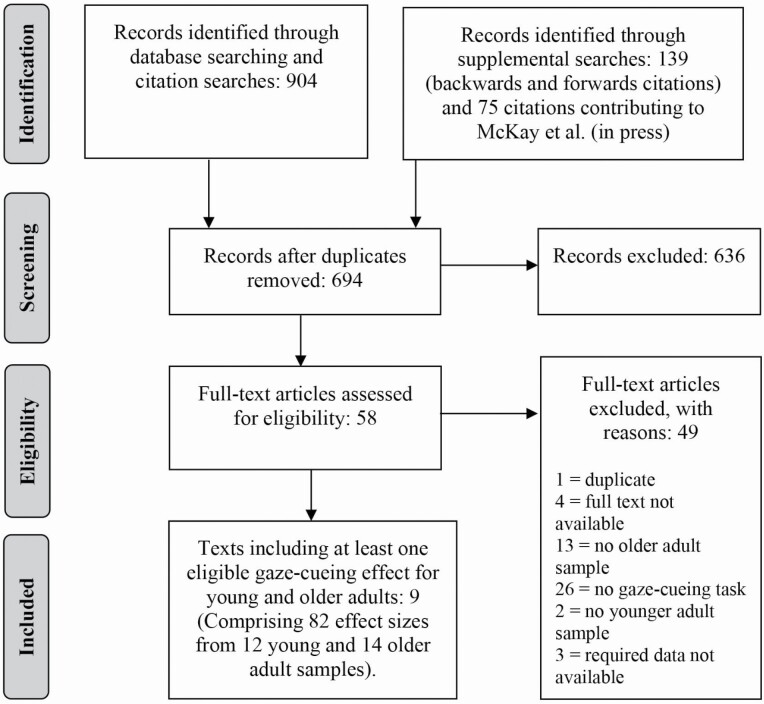
Preferred Reporting Items for Systematic Reviews and Meta-Analyses (PRISMA) flow diagram illustrating the study screening and selection process (as specified by Moher et al., 2009).

Figure 2 provides a flow diagram illustrating the study selection process. Studies were included if they were available in English, included both a healthy older adult sample and a healthy younger adult sample, measured response times to peripheral targets following the presentation of a central averted gaze cue, and provided or made available the required data for effect size calculation. The study selection process is detailed in Supplement 1. Comprehensive database searches were conducted in May 2021 using the terms (Ageing OR Aging OR Older) in combination with (Gaze OR Gazing) in combination with (Cues OR Cueing OR Cuing OR Cued) and supplemental backward and forward citations were also conducted. Abstract and full-text screening were completed in duplicate. Specific reasons for any exclusion at full-text review are provided within Supplement 1.

### Statistical Approaches

#### Effect size calculation

Standardized mean change effect sizes (*SMC*s) were calculated in R “metafor” (Viechtbauer, 2010). The correlation between gaze-cued and gaze-miscued trials was imputed (see Supplement 2 for detail). We calculated *SMC*s using the raw score standardization approach (as specified in Becker, 1988). Meta-analytic effect sizes were calculated by integrating the *SMC*s using multilevel modeling in R “metafor” to retain statistical power that would be lost using alternative methods of dealing with multilevel data (e.g., taking one effect size per sample). Random effects models were used.

#### Moderator analyses

To determine whether there were age differences in gaze-cueing, we assessed moderation of gaze-cueing effects by age group (young vs older adults) among all effects and then among effects associated with predictive and nonpredictive gaze-cueing tasks separately (Author Note 2). We then assessed the age effects after controlling for the cue-target stimulus onset asynchrony (SOA) which refers to the length of time in milliseconds between the onset of the gaze cue and the onset of the target, task type which refers to whether participants had to detect, localize, or categorize the target stimuli, and face type which refers to whether the gaze cues were schematic drawings, computer-generated images, or images of real faces. These specific variables were chosen because of previously shown age-related effects. SOA is controlled for given previously uncovered age differences in processing speed and sustained attention (Albinet et al., 2012; Staub et al., 2013). Task type is controlled for given previously established age-related changes in working memory capacity and visual search performance (Hartshorne & Germine, 2015; Plude et al., 1994). Face type was controlled for given that responsiveness or engagement with tasks that vary in ecological validity may differ across the life span due to age-related changes in motivation (in line with Carstensen, 1992).

#### Age effect sizes

Central to differentiating between the visual attention and cognitive decline models is determining whether predictive or nonpredictive gaze-cueing specific age differences are larger. A meta-analytic method that can test for these types of interaction effects is not yet available, but what we are able to do is calculate standardized mean *differences*, Cohen’s *d*s, at the effect size level. In this case, the mean differences being compared are the meta-analytic means; the standard deviations are those of individual effects around the meta-analytic mean effects within each group; and the sample sizes are the number of effects available for each group (effect sizes [ESs]). These were calculated using the online software Psychometrica (Lenhard & Lenhard, 2016); wherein, a Hedges *g* correction for small samples is also automatically applied within the calculation. For this reason, these effect sizes are referred to as *g*s.

To calculate the magnitude of the age effects associated with the *t* tests arising from the follow-up multiple moderator analyses, the formula presented in Lakens (2013) was used (see Equation 1) but again because we wanted to quantify a difference between effect sizes rather than individuals, this was calculated at the effect size level. To this end, we used the *t* test associated with the age-specific effect size effect, and as before the sample sizes were the respective ESs. A Hedges *g* correction was applied as specified in Lakens (2013; Equation 2).


$$\begin{array}{l} d=t\sqrt{\frac{1}{ES1}+\;\frac{1}{ES2}} \end{array}$$ (1)

$$\begin{array}{l} g=d\times \left(1-\frac{3}{4(ES1+ES2)-9}\right) \end{array}$$ (2)

### Data Extraction

At the effect size level, the mean and standard deviation reaction times in gaze-cued and gaze-miscued trials, the correlation between reaction times in gaze-cued and gaze-miscued trials (where available), whether the gaze cues were predictive or nonpredictive, the SOA used, the task type, and the face type were extracted. At the sample level, the post-data-reduction number of participants, number of female participants, and participant mean age were extracted as well as the participant age group, and the country in which the study was conducted. For the publication bias analyses, publication status, and the *p* value associated with the first test of age differences in gaze-cueing effects with a significant (α < 0.05) result for each study were extracted (along with other information required for the *p*-curve table).

#### Interrater reliability

To assess the interrater reliability of the data extraction procedure, the second author performed data extraction of moderator and control variables (task type, face type, SOAs used, and age group) for a randomly selected subset of 50% of the samples. Interrater reliability was assessed using the R package irr (Gamer et al., 2019). For all four double-coded variables, percentage agreement was 100% (all κs = 1).

## Results

### Sample Characteristics

We identified 82 gaze-cueing effects (*k* = 26, *N* = 919 participants). Of these, 37 were associated with young adults (*k* = 12, *n* = 438) and 45 were associated with older adults (*k* = 14, *n* = 481). Ten younger adult and 11 older adult samples reported the mean age and number of female participants post-data reduction. The mean of the mean sample ages was 20.85 years for younger adults and 71.66 years for the older adults. The proportion of female participants was 71.20% for younger adults and 68.25% for older adults. All primary study effect sizes and the sample and task information associated with each effect size are provided in Supplement 3. Table 1 reports all meta-analytic effect sizes and associated statistics.

**Table 1.** Meta-Analytic Gaze-Cueing Effect Sizes and Associated Relevant Statistics Overall and for Predictive and Nonpredictive Cues Separately for Younger and Older Adult Samples and Results for Moderation by Age Group

	Effect size information										Moderation by age group			
	*k*	*ES*	*N*	*SMC*	*SE*	*t*	*df*	*p*	95% CI	*Q*	*F*	*df*	*p*	*g*
Overall														
Young adults	12	37	438	0.31	0.09	3.63	36	<.001	[0.14, 0.49]	316.42***				
Older adults	14	45	481	0.10	0.01	8.07	44	<.001	[0.08, 0.13]	82.07***				
											7.72	1, 80	.007	−0.59
Predictive														
Young adults	4	8	172	0.70	0.09	7.99	7	<.001	[0.50, 0.91]	14.19*				
Older adults	4	8	159	0.12	0.02	5.22	7	.001	[0.06, 0.17]	3.79				
											94.48	1, 14	<.001	−3.24
Nonpredictive														
Young adults	9	29	307	0.18	0.02	7.82	28	<.001	[0.13, 0.22]	84.93***				
Older adults	11	37	356	0.10	0.02	6.54	36	<.001	[0.07, 0.13]	77.31***				
											8.41	1, 64	.005	−0.78

*Notes*: CI = confidence interval. *k*, *ES*, and *N* are the number of samples, effect sizes, and participants, respectively. *SMC* is the standardized mean change gaze-cueing effect. *Q* is the statistic associated with the test for heterogeneity. *Q*(*df*) = *t*(*df*).

***p < .001. *p < .05.

### Meta-Analytic Age Effects

**Figure 3.**
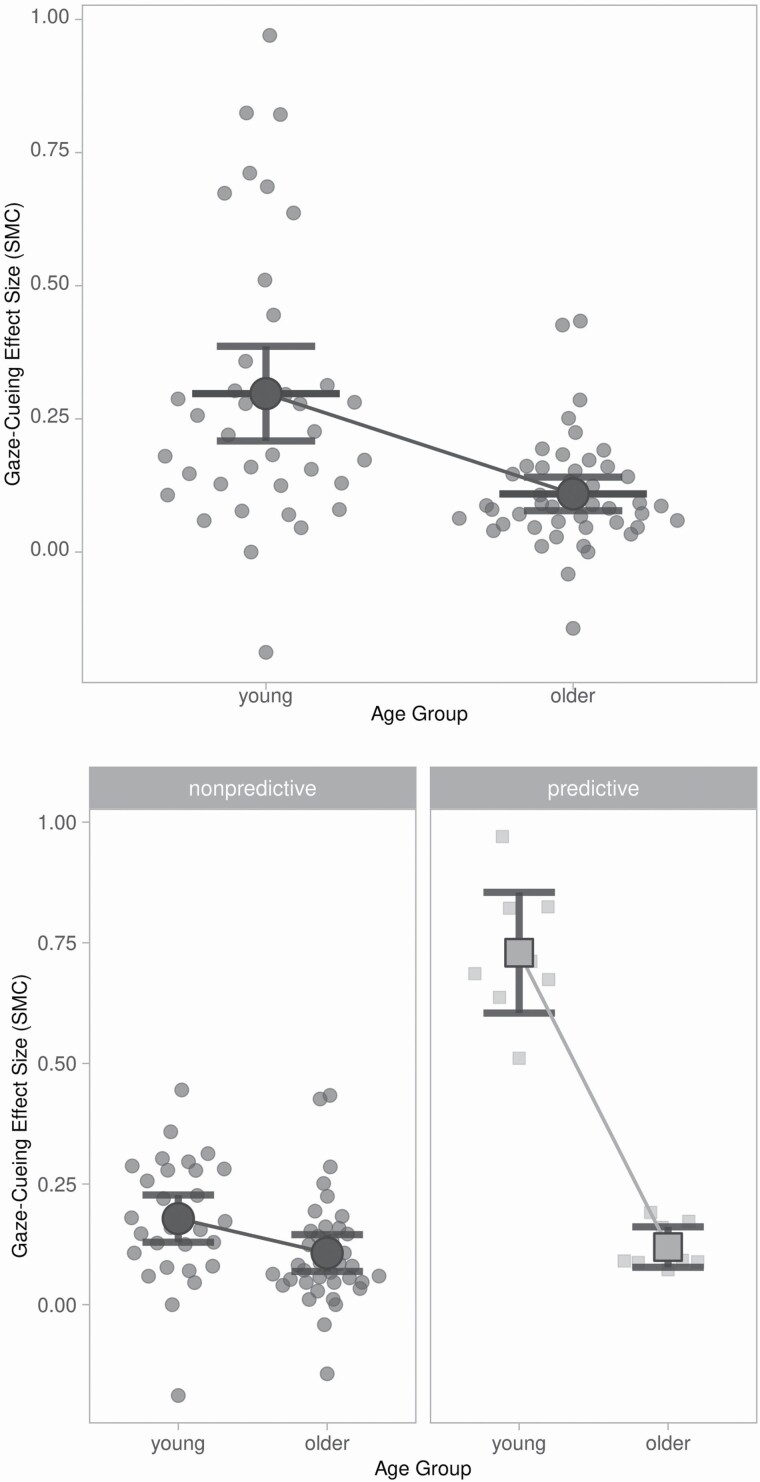
Gaze-cueing effects by age group overall (top) and for predictive (bottom left) and nonpredictive (bottom right) cues separately. *Note*: The mean effect size for each age group is represented by the larger superordinate shapes that each have an associated pair of error bars. Error bars represent the 95% confidence interval around the mean effect size for each age group. Figure created using SuperPlotsOfData (Goedhart, 2021).

For a visualization of the results, see Figure 3. Both younger and older adults had significant overall gaze-cueing effects. However, older adults had a significantly reduced gaze-cueing effect relative to younger adults, *F*(1, 80) = 7.72, *p* = .007, *g* = −0.59. This age effect was retained in a multiple moderator model controlling for SOA, task type, and face type, *t*(75) = 3.07, *p* = .003, *g* = −0.67. Significant heterogeneity remained, *QE*(75) = 329.51, *p* < .001.

For predictive gaze-cueing tasks, older and younger adults had significant gaze-cueing effects. However, in predictive gaze-cueing tasks, older adults had significantly reduced gaze-cueing relative to younger adults, *F*(1, 14) = 94.48, *p* < .001, *g* = −3.24. In the multiple moderator model controlling for SOA (all effects in this analysis were localization tasks with real face image cues), this age effect remained significant, *t*(13) = 7.88, *p* < .001, *g* = 3.73. Remaining heterogeneity was not significant, *QE*(13) = 16.65, *p* = .216.

For nonpredictive gaze-cueing tasks, older and younger adults had significant gaze-cueing effects. However, in nonpredictive gaze-cueing tasks, older adults had significantly reduced gaze-cueing relative to younger adults, *F*(1, 64) = 8.41, *p* = .005, *g* = −0.78. This age effect was retained in a multiple moderator model controlling for SOA, task type, and face type, *t*(59) = 3.66, *p <* .001. Significant heterogeneity remained, *QE*(59) = 111.96, *p* < .001, *g* = 0.90.

### Publication Bias Analyses

Rogers and Pustejovsky’s (2021) multilevel and robust variance estimation methods were used to assess funnel plot asymmetry among the full data set to establish whether there was evidence of bias within the extracted gaze-cueing effects. A *p*-curve analysis of the *p* values associated with tests of age differences in gaze-cueing effects was conducted to assess whether there was evidence of bias according to the significance of age effects on gaze-cueing. The *p*-curve analysis is an assessment of the distribution of significant *p* values across a literature. It is not a multilevel test and so only one *p* value was extracted per study. For consistency, this was always the *p* value associated with the first-reported test of age differences in gaze-cueing effects that was significant (*p* < .05). These *p* values were then subjected to a *p*-curve analysis via the *p*-curve online app (http://www.p-curve.com/app4/). It is assumed that the distribution of significant *p* values for a real effect will be right-skewed (see Simonsohn et al., 2014 for a detailed explanation).

**Figure 4.**
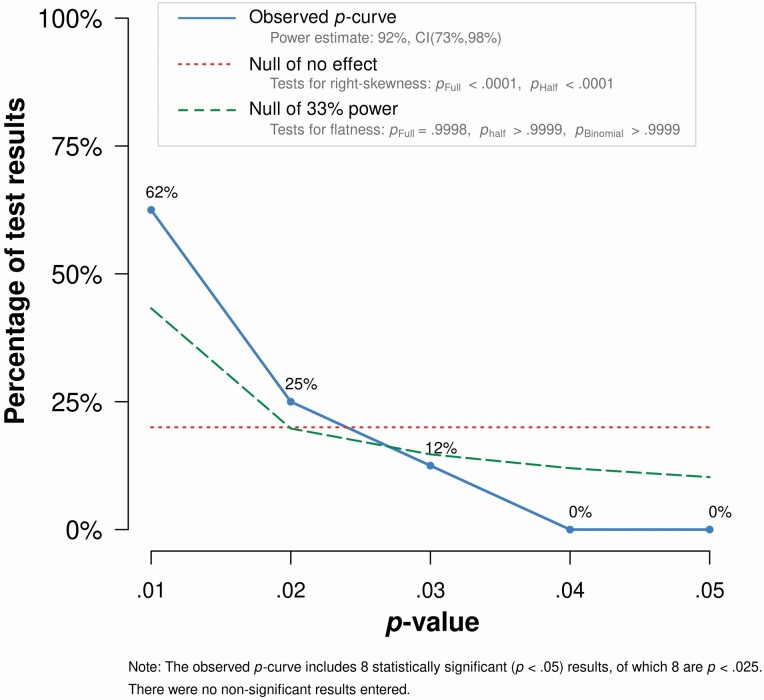
*p*-Curve for the set of first-reported significant age group × gaze-cueing effect interactions.

We did not identify any evidence of publication bias. The Egger’s regression tests using robust variance estimation and multilevel modeling both returned nonsignificant results: β = 6.79, *SE* = 4.03, *p* = .071 and β = 5.29, *SE* = 1.18, *p* = .070, respectively, indicating no statistically significant asymmetry in the distribution of effect sizes and therefore no evidence of selective reporting at the effect size level. Supplement 4 provides the *p*-Curve Disclosure Table. As can be observed in Figure 4, the distribution of *p* values appears to be right-skewed. The continuous test of the *p*-curve using Stouffer’s method was significant for both the full-curve assessment, *Z* = −5.78, *p* < .001, and the half-curve assessment, *Z* = −4.18, *p* < .001, confirming that the distribution of significant age effects on gaze-cueing was significantly right-skewed for *p*s < .05, and *p*s < .025, respectively (although note all *p*s were <.025 and both results are reported only to be consistent with reporting standards for *p*-curves). In conclusion then, the results of the present *p*-curve analysis suggest that the observed *age effects* on gaze-cueing from these studies is likewise not likely to be a result of selective reporting (e.g., *p*-hacking, publication bias).

### Outlier and Sensitivity Analyses

We conducted the key statistical analyses on three subsets of effect sizes: namely, the overall sample of gaze-cueing effects, the sample of gaze-cueing effects associated with predictive gaze-cueing tasks, and the sample of gaze-cueing effects associated with nonpredictive gaze-cueing tasks. Within each of these three subsets, we assessed for the presence of statistical outliers by calculating the standardized residuals in a post hoc analysis in light of a visual inspection of the distribution of effect sizes raising the possibility of outliers. Scores exceeding ±2.24 were regarded as extreme (Aguinis et al., 2013). Where outliers were detected, we conducted sensitivity analyses wherein we recomputed the significant moderation by age effects uncovered within those data sets after excluding the outlying effect sizes.

Within the overall data set, three positive outliers were detected (Slessor et al., 2008, effect ID 66; Slessor et al., 2008, effect ID 67; and Slessor et al., 2016, effect ID 77). In a sensitivity analysis excluding these three effect sizes, the moderating effect of age on the overall data set was retained, *F*(1, 77) = 6.97, *p* = .010. Within the predictive cues only data set, no outliers were detected. Within the nonpredictive cues only data set, two positive outliers were detected (Bailey et al., 2014, effect ID 8; and Slessor et al., 2016, effect ID 74). In a sensitivity analysis excluding these two effect sizes, the moderating effect of age on the nonpredictive data set was retained, *F*(1, 62) = 9.74, *p* = .003.

## Discussion

These data provide compelling evidence for an overall age-related reduction in gaze-cued attention, with an age-related reduction evident for both predictive and nonpredictive gaze cues. However, the magnitude of this age effect was substantially larger for predictive relative to nonpredictive cues with an enhancement of gaze-cueing for predictive gaze cues evident only for younger, but not older adults. These results therefore clearly align with the cognitive decline model of social-cognitive aging and therefore provide important insights not only into the nature and magnitude of gaze-cueing age effects, but also into the potential mechanisms that contribute to this age-related reduction.

This finding that predictive gaze cues seem to “boost” younger adults’ gaze-cueing is consistent with Olk and Kingstone’s (2015) proposal that volitional effects—arising from predictive cues that produce reflexive orienting in and of themselves—have an additive effect on attentional cueing. That is, across both predictive and nonpredictive tasks, reflexive stimulus-driven attention should occur, given the presence of the stimulus in all trials, irrespective of predictiveness. However, when cues are predictive, additional volitional top-down orienting occurs which “boosts” attentional cueing effects. The current findings suggest that this “boost” elicited by predictive cues occurs for younger but not older adults, in line with the cognitive decline model of social-cognitive aging.

The findings presented here are in line with broader literature focused on selective visual attention across the life span. Reflexive orienting *to* nonsocial sudden onset stimuli is known to be relatively spared by aging (see Plude et al., 1994 for a review). Our findings show that reflexive orienting *via* social gaze cue stimuli (generally considered a distinct orienting process) is likewise relatively spared. Our finding that volitional gaze-cued attention is particularly disrupted meaningfully extends current understanding of attention and aging then by showing that older adults demonstrate disruption to their capacity to strategically use social gaze cues. Specifically, these findings suggest that visual selective attention is broadly affected by aging, such that there are age-related losses, not only in nonsocial, but also in social visual attentional orienting processes.

With respect to other age-related attentional changes that might have contributed to the age-related reductions in gaze-cueing identified here, it seems unlikely that older adults are simply better at ignoring the gaze cue stimuli compared to younger adults. This is because prior research shows that there is a decreased capacity to exert inhibitory processing later in life (Plude et al., 1994) as well as age-related difficulties is disengaging attention once it has been engaged (Polden et al., 2020). Less clear however is whether our findings might be explained by age-related general slowing—a phenomenon that results in longer reaction times for older compared to younger adults (Verhaeghen & Cerella, 2002). What we found was that the gaze-cueing-indicative *difference* between response times emerged for but was *smaller* for older compared to younger adults, a finding that is not in itself diagnostic of slowed attention. In saying that, our findings are not diagnostic of the effect *not* being driven by slowed attention either. The possibility that older adults simply have a temporally shifted pattern of gaze-cued attention such that their gaze-cueing effect peaks at a later SOA compared to young adults should be directly tested in future research. Existing research on age effects in nonsocial reflexive visual attentional orienting fails to provide a clear answer to this question. In some studies, a smaller *inhibition of return* effect (reversed cueing effects at later SOAs which represent the waning of attention at the oriented-to-location over time) has been identified, suggesting that older adults may indeed have a rightward-shifted pattern of attentional cueing across time. However, other studies have reported the opposite (a larger inhibition of return effect) or no age effect on inhibition of return (see Erel & Levy, 2016 for a review). To establish whether and, if so, how general slowing might contribute to age effects in gaze-cued attention, future investigations of gaze-cued attention across the life span now need to extend their work to include late SOAs.

Gaze-cued attention is thought to be one of the core mechanisms that, in combination with other social-cognitive mechanisms, underpins our capacity to develop and maintain social connections (Stephenson et al., 2021). Because social connection is a critical predictor of many healthy aging outcomes (Livingston et al., 2020), it is important to understand how the social-cognitive processes that subserve our capacity to connect with others are affected by normal adult aging. This meta-analysis provided clear evidence of an age-related reduction in reflexive and volitional gaze-cued attention; however, as will be discussed, further research is needed to address potential implications of this reduction for other higher-order aspects of social cognition such as theory of mind, as well as, and perhaps more critically, older adults’ real-life social functioning. Indeed, based on the present literature, we simply do not know whether age-related reductions in gaze-cueing play any role in older adults’ social relationships.

Although older adults typically report comparable and sometimes greater levels of socioemotional well-being relative to younger adults (Mather, 2016), older adults typically perform more poorly than younger adults on laboratory tests of social-cognitive function. Here, the most common finding has been of age-related decline in many core social-cognitive skills, such as problems recognizing others’ emotional expressions (Hayes et al., 2020) and a reduced ability to understand the mental states of others (Henry et al., 2013). An important next step will be to determine whether and how age-related decline in gaze-cued attention might be related to these other age-related social-cognitive losses. There has been no previous direct test of this question, but theoretically, there are strong grounds for predicting that such an association should exist. For instance, existing theories of social cognition—namely the mindreading model and its various iterations—posit that gaze-cued attention is a core lower-order social-cognitive mechanism that subserves the development of higher-order social-cognitive mechanisms such as empathy and theory of mind (see Emery, 2000; Stephenson et al., 2021 for reviews). This suggests that future research should consider age-related reductions in gaze-cued attention as a factor that potentially contributes to age-related losses in higher-order social-cognitive abilities.

Another important consideration for future research is to establish whether the age-related reductions in gaze-cueing effects identified in this meta-analysis generalize to more realistic social stimuli. As is the case with much of the broader social-cognitive aging literature, all gaze-cueing paradigms that contributed to this review relied on quite artificial stimuli (these were images of strangers and cartoon faces). At most, the illusion of biological motion was induced via the presentation of multiple slightly different images wherein the gaze cue shifts from direct to averted across the trial (e.g., as in Dalmaso et al., 2015; Nagy et al., 2020; Slessor et al., 2008). Most studies, however, presented only a single static averted gaze cue (e.g., as in Bailey et al., 2014; Deroche et al., 2016; Gayzur et al., 2014; Slessor et al., 2016). These methodological approaches provide excellent laboratory control but raise obvious questions about whether the same age effects would emerge when more realistic stimuli and methods are used. It is possible that age effects could be reduced or possibly even eliminated in situations of high personal relevance. Extensive literature now provides evidence of motivational shifts with aging, whereby personally relevant and meaningful goals are increasingly prioritized (see Carstensen, 2021). If older adults are more motivated to engage with people they know and care about, this might quite fundamentally alter attention and responsiveness to eye gaze cues. We therefore consider the development of more ecologically valid approaches to be the next important step in this literature. This would more directly speak to whether the age-related reductions in gaze-cueing, which this meta-analysis reveals to be a robust feature of older adults’ performance in laboratory settings, have implications for their real-life social functioning.

The presence of remaining heterogeneity among the gaze-cueing effect sizes after accounting for age group that we found here for social attentional cueing suggests that gaze-cueing effects are modulated by other variables including, for example, context (see Dalmaso et al., 2020 for a review), or cue-and-task features (see McKay et al., 2021 for a review). We suggest that potential moderator variables of the age effect on gaze-cueing should be investigated in future research. As discussed above, particularly critical potential moderators include SOA and ecological validity.

### Limitations

In terms of limitations, it needs to be acknowledged that there were a relatively small number of contributing studies; however, the number of contributing effect sizes was large, and the results are nonetheless clear, a conclusion reflected in the relatively narrow confidence intervals for all meta-analytic effects here.

## Conclusion

To summarize then, this meta-analysis provides the clearest evidence to date that gaze-cued attention, a central social-cognitive process, is associated with a decline in magnitude in older adulthood. Future studies are now needed to interrogate possible mechanisms driving this effect and to establish how these age differences in gaze-cued attention might affect on older adults’ real-life social functioning.

## Supplementary Material

gbac052_suppl_Supplementary_Material

## Author Note

1. Note that data were insufficient to conduct all preregistered aims and analyses. All data and analysis scripts are available on the Open Science Framework page.

2. Note that primary studies to date do not report *age effects* in gaze-cueing magnitudes but rather gaze cue validity × age group membership interactions, making a meta-regression assessing moderation of the age effect by other variables impossible at this time.
