# Supplementary material for: How Does Aging Affect Social Attention? A Test of Competing Theories Using Multilevel Meta-Analysis
Source: J Gerontol B Psychol Sci Soc Sci. 2022 Mar 12;77(8):1454–63. doi: 10.1093/geronb/gbac052 (PMC9371458; doi:10.1093/geronb/gbac052)
Supplement: gbac052_suppl_Supplementary_Material [file gbac052_suppl_supplementary_material.pdf]

How Does Ageing Affect Social Attention? A Test of Competing Theories using Multi-Level Meta-Analysis

Supplemental Material

Table of Contents

Supplement 1: Study Selection Processes 3

Supplement 1a. Description of the Study Selection Process 3

Supplement 1b. Specific Reasons for the Exclusion of Citations that were Full Text Reviewed 4

Supplement 1c. Citations for Final Set of Included Papers/Theses 6

Supplement 2: Details Regarding Effect Size Calculation Correlation Imputation 7

Supplement 3: All Included Gaze-Cueing Effects along with their Corresponding Sample and Task Characteristics 8

Supplement 4: *P*-Curve Disclosure Table 13

Supplement 1: Study Selection Processes

Supplement 1a. Description of the Study Selection Process

Database Searches

A comprehensive systematic review was conducted as follows with the aim of ensuring the best chance of finding all relevant studies meeting the inclusion criteria. The study selection process is illustrated in Figure 1. In May 2021, title and abstract searches of Web of Science, PSYCINFO, PUBMED, and Scopus were completed using the terms (Ageing OR Aging OR Older) in combination with (Gaze OR Gazing) in combination with (Cues OR Cueing OR Cuing OR Cued). A total of 904 citations were uncovered. After removing 301 duplicates, the remaining 603 citations were screened independently by the first and fourth authors. This was done in duplication to ensure no eligible studies were excluded mistakenly. At this stage, citations were excluded for clear ineligibility. Any citation that at least one screener recommended full text review for was sent forward to the full text review stage. In total, 56 citations were sent forward to the full-text-review stage and reviewed against the inclusion criteria independently in duplicate by two reviewers (and assessed by a third reviewer whose decision was final if a disagreement arose). Forty-nine texts were excluded due to ineligibility against the inclusion criteria (see Supplemental Material 1 for text-specific reasons). In the end then, through database searches, 7 texts were determined to have at least one eligible gaze-cueing effect for younger and older adult samples (Bailey et al., 2014; Deroche et al., 2016; Gayzur et al., 2014; Nagy et al., 2020; Slessor et al., 2010; 2008; 2016).

Additional Supplementary Searches

To ensure recovery of all eligible texts, the first author completed forwards citations searches of 12 specific texts initially identified as eligible through the database searches as well as a backwards citations search of all papers cited in a recent review of gaze-cueing effects (McKay et al., *under review*). These additional searches can be considered an additional supplemental search completed with the intention of ensuring any eligible texts not picked up in the database searches would be identified and were preregistered prior to data extraction. PSYCinfo was used to retrieve the cited by list for each paper on 24 July, 2021. Citations were considered *possibly relevant* if the title mentioned use of older adult or lifespan samples or a clinical sample that would reasonably include older adult controls (e.g., dementia, stroke, etc.) as well as anything related to eye gaze. In total, 139 citations were identified. Of these, 96 were unique and retained for screening (see supplemental material for a list). Of these, 34 were considered potentially relevant. After cross checkeing with those already screened during the database search, 16 were novel citations. The abstracts of these 16 novel citations full texts were screened by the first author. One was determined to be eligible (Dalmaso et al., 2015). The backwards citations search of McKay et al. (*under review*) revealed one eligible novel citation (Insch, 2013). In the end then, a total of 9 eligible papers and theses were identified and included in the meta-analysis.

Supplement 1b. Specific Reasons for the Exclusion of Citations that were Full Text Reviewed

Burra, N., Kerzel, D., de Gelder, B., & Pegna, A. J. (2014).	No younger adult sample
Insch, P. M., Slessor, G., Warrington, J., & Phillips, L. H. (2017).	No younger adult sample
Kuhn, G., Pagano, A., Maani, S., & Bunce, D. (2015).	Data not available
Petrican, R. (2012).	Data not available
Petrican, R., English, T., Gross, J. J., Grady, C., Hai, T., & Moscovitch, M. (2012).	Data not available
Akiyama, T., Kato, M., Muramatsu, T., Maeda, T., Tsunekatsu, H., & Kashima, H. (2008).	No older adult sample
Akiyama, T., Kato, M., Muramatsu, T., Umeda, S., Saito, F., & Kashima, H. (2007).	No older adult sample
Boll, S., Bartholomaeus, M., Peter, U., Lupke, U., & Gamer, M. (2016).	No older adult sample
Ciardo, F., De Angelis, J., Marino, B. F. M., Actis-Grosso, R., & Ricciardelli, P. (2021).	No older adult sample
Dalmaso, M., Edwards, S. G., & Bayliss, A. P. (2015).	No older adult sample
Magnée, M. J. C. M., Kahn, R. S., Cahn, W., & Kemner, C. (2011).	No older adult sample
Marotta, A., Casagrande, M., Rosa, C., Maccari, L., Berloca, B., & Pasini, A. (2014).	No older adult sample
Okada, T., Sato, W., Kubota, Y., Usui, K., Inoue, Y., Murai, T., Hayashi, T., & Toichi, M. (2008).	No older adult sample
Redcay, E., Kleiner, M., & Saxe, R. (2012).	No older adult sample
Rigato, S., Menon, E., Di Gangi, V., George, N., & Farroni, T. (2013).	No older adult sample
Sato, W., Uono, S., Okada, T., & Toichi, M. (2010).	No older adult sample
Saunders, K. E. A., Goodwin, G. M., & Rogers, R. D. (2015).	No older adult sample
Uono, S., Sato, W., & Toichi, M. (2009).	No older adult sample
Berchio, C., Küng, A., Kumar, S., Cordera, P., Dayer, A. G., Aubry, J., Michel, C. M., & Piguet, C. (2019).	No gaze-cueing task
Bertsch, K., Gamer, M., Schmidt, B., Schmidinger, I., Walther, S., Kästel, T., Schnell, K., Büchel, C., Domes, G., & Herpertz, S. C. (2013).	No gaze-cueing task
Campbell, A., Murray, J. E., Atkinson, L., & Ruffman, T. (2017).	No gaze-cueing task
Caruana, N., Ham, H. S., Brock, J., Woolgar, A., Kloth, N., Palermo, R., & McArthur, G. (2017).	No gaze-cueing task
Fernandes, E. G., Phillips, L. H., Slessor, G., & Tatler, B. W. (2021).	No gaze-cueing task
Freebody, S., & Kuhn, G. (2016).	No gaze-cueing task
Gaál, Z. A., Nagy, B., File, D., & Czigler, I. (2020).	No gaze-cueing task
Gazes, Y., Habeck, C., O'Shea, D., Razlighi, Q. R., Steffener, J., Stern, Y. (2015).	No gaze-cueing task

Grainger, S. A., Henry, J. D., Phillips, L. H., Vanman, E. J., & Allen, R. (2017).	No gaze-cueing task
Grainger, S. A., Steinvik, H. R., Henry, J. D., & Phillips, L. H. (2019).	No gaze-cueing task
Jimenez, S., Hollands, M., Palmisano, S., Kim, J., Markoulli, M., McAndrew, D., Stamenkovic, A., Walsh, J., Bos, S., & Stapley, P.J. (2016).	No gaze-cueing task
Morehouse, S. A. (2000).	No gaze-cueing task
Noh, S. R., Lohani, M., & Isaacowitz, D. M. (2011).	No gaze-cueing task
Ossenfort, K. (2021).	No gaze-cueing task
Redcay, E., Ludlum, R.S., Velnoskey, K.R., & Kanwal, S. (2016).	No gaze-cueing task
Richard-Mornas, A., Borg, C., Klein-Koerkamp, Y., Paignon, A., Hot, P., Thomas-Antérion, C. (2012).	No gaze-cueing task
Seitz, K. I., Leitenstorfer, J., Krauch, M., Hillmann, K., Boll, S., Ueltzhoeffer, K., Neukel, C., Kleindienst, N., Herpertz, S. C., & Bertsch, K. (2021).	No gaze-cueing task
Seya, Y., Tsutsui, K., Watanabe, K., & Kimura, K. (2012).	No gaze-cueing task
Sheldon, S., Quint, J., Hecht, H., & Bowers, A.R. (2014).	No gaze-cueing task
Slessor, G., Phillips, L.H., & Bull, R. (2010).	No gaze-cueing task
Slessor, G., Phillips, L.H., & Bull, R. (2010).	No gaze-cueing task
Slessor, G., Riby, D. M., & Finnerty, A. N. (2013).	No gaze-cueing task
Vicaria, I. M., Bernieri, F. J., & Isaacowitz, D. M. (2015).	No gaze-cueing task
Vlamings, P. H. J. M., Stayder, J. E. A., van Son, I. A. M., & Mottron, L. (2005).	No gaze-cueing task
Xing, C. (2009).	No gaze-cueing task
Ziaei, M., Burianová, H., von Hippel, W., Ebner, N.C., Phillips, L.H., & Henry, J.D. (2016).	No gaze-cueing task
Slessor, G., Laird, G., Phillips, L. H., Bull, R., & Filippou, D. (2010).	Duplicate citation not detected at abstract screening
Tofield, M. I., & Wann, J. P. (2002).	Full text not available
Chen, S. R., & Li, J. L. (2019).	Full text not available
Gayzur, N., Langley, L., Wyman, S. V., Saville, A., & Friesen, C. (2011).	Full text not available
McCade, D. L., Guastella, A. J., Chen, N. T. M., Lewis, S. J. G., & Naismith, S. L.	Full text not available

Supplement 1c. Citations for Final Set of Included Papers

Bailey, P. E., Slessor, G., Rendell, P. G., Bennetts, R. J., Campbell, A., & Ruffman, T. (2014). Age differences in conscious versus subconscious social perception: The influence of face age and valence on gaze following. *Psychology and Aging, 29*(3), 491-502. http://doi.org/10.1037/a0036249

Dalmaso, M., Castelli, L., Priftis, K., Buccheri, M., Primon, D., Tronco, S., & Galfano, G. (2015). Space-based and object-centred gaze cuing of attention in right hemisphere-damaged patients. *Frontiers in Psychology, 6*, 1119. http://doi.org/10.3389/fpsyg.2015.01119

Deroche, T., Castanier, C., Perrot, A., Hartley, A. (2016). Joint attention is slowed in older adults. *Experimental Aging Research, 42*(2), 144-150. http://doi.org/10.1080/0361073X.2016.1132826

Gayzur, N. D., Langley, L. K., Kelland, C., Wyman, S. V., Saville, A. L., Ciernia, A. T., & Padmanabhan, G. (2014). Reflexive orienting in response to short- and long-duration gaze cues in young, young-old, and old-old adults. *Attention, Perception, and Psychophysics, 76*, 407-419. http://doi.org/10.3758/s13414-013-0554-6

Insch, P. M. (2013). Exploring the impact of aging and dementia on the precursors to theory of mind (unpublished doctoral thesis). University of Aberdeen, Scotland used predictive gaze cues.

Nagy, B., Czigler, I., File, D., & Gaál, Z. A. (2020). Can irrelevant but salient visual cues compensate for the age-related decline in cognitive conflict resolution?—An ERP study. *PLoS ONE, 15*(5). http://doi.org/10.1371/journal.pone.0233496

Slessor, G., Laird, G., Phillips, L. H., Bull, R., & Filippou, D. (2010). Age-related differences in gaze following: Does the age of the face matter? *Journal of Gerontology: Psychological Sciences, 65B*(5), 536-541. http://doi.org/10.1093/geronb/gbq038

Slessor, G., Phillips, L. H., & Bull, R. (2008). Age-related declines in basic social perception: Evidence from tasks assessing eye-gaze processing. *Psychology and Aging, 23*(4), 812-822. http://doi.org/10.1037/a0014348

Slessor, G., Venturini, C., Bonny, E. J., Insch, P. M., Rokaszewicz, A., & Finnerty, A. N. (2016). Specificity of age-related differences in eye-gaze following: Evidence from social and nonsocial stimuli. *Journals of Gerontology: Psychological Sciences, 71*(1), 11-22. http://doi.org/10.1093/geronb/gbu088

Supplement 2: Details Regarding Effect Size Calculation Correlation Imputation

We expected to have to impute the correlation between gaze-cued and gaze-miscued trials due to a lack of reporting of this variable. For this, we extracted the gaze-cued to gaze-miscued correlation wherever possible and computed a multi-level meta-analytic correlation coefficient for use as a representative correlation for imputation where this value is missing. We were able to extract valid-invalid correlations associated with 20 gaze-cueing effects from six samples comprising 210 participants. The meta-analytic point estimate of the valid-invalid correlation was r = .93, 95% CI [.72, .98]. We used the correlation to 6 decimal places for precision. It was r = .929433. This is a typical magnitude for a pre-post correlation (Estrada et al., 2019).

Estrada, E., Ferrer, E., & Pardo, A. (2019). Statistics for evaluating pre-post change: Relation between change in the distribution center and change in the individual scores. *Frontiers in Psychology, 9*(2696). http://doi.org/10.3389/fpsyg.2018.02696

Supplement 3. All Included Gaze-Cueing Effects along with their Corresponding Sample and Task Characteristics

Citation	Sample Characteristics[1]						Effect Characteristics and Moderators of Interest						
**Author/s (year)**	***k* ID**	***N***	**Female *N***	**Age Group**	**Mean Age**	**Country Study Conducted In**	***ES* ID**	**Predictiveness**	**Face Type**	**Cue-Target SOA (ms)**	**Task Type[2]**	**Emotion**	**Effect Size *SMC***
Bailey et al. (2014)	1	31	nr	young	nr	Australia	1	nonpredictive	real	200	local	neutral	0.16
Bailey et al. (2014)	1	31	nr	young	nr	Australia	2	nonpredictive	real	200	local	neutral	0.08
Bailey et al. (2014)	1	31	nr	young	nr	Australia	3	nonpredictive	real	200	local	neutral	0.16
Bailey et al. (2014)	1	31	nr	young	nr	Australia	4	nonpredictive	real	200	local	neutral	0.30
Bailey et al. (2014)	2	31	nr	older	nr	Australia	5	nonpredictive	real	200	local	neutral	0.06
Bailey et al. (2014)	2	31	nr	older	nr	Australia	6	nonpredictive	real	200	local	neutral	0.09
Bailey et al. (2014)	2	31	nr	older	nr	Australia	7	nonpredictive	real	200	local	neutral	-0.04
Bailey et al. (2014)	2	31	nr	older	nr	Australia	8	nonpredictive	real	200	local	neutral	0.43
Bailey et al. (2014)	3	32	nr	young	nr	Australia	9	nonpredictive	real	200	local	happy	0.26
Bailey et al. (2014)	3	32	nr	young	nr	Australia	10	nonpredictive	real	200	local	fearful	0.22
Bailey et al. (2014)	3	32	nr	young	nr	Australia	11	nonpredictive	real	200	local	happy	0.28
Bailey et al. (2014)	3	32	nr	young	nr	Australia	12	nonpredictive	real	200	local	fearful	0.18
Bailey et al. (2014)	3	32	nr	young	nr	Australia	13	nonpredictive	real	200	local	happy	0.08
Bailey et al. (2014)	3	32	nr	young	nr	Australia	14	nonpredictive	real	200	local	fearful	0.06
Bailey et al. (2014)	3	32	nr	young	nr	Australia	15	nonpredictive	real	200	local	happy	0.30
Bailey et al. (2014)	3	32	nr	young	nr	Australia	16	nonpredictive	real	200	local	fearful	0.13
Bailey et al. (2014)	4	30	nr	older	nr	Australia	17	nonpredictive	real	200	local	happy	0.03
Bailey et al. (2014)	4	30	nr	older	nr	Australia	18	nonpredictive	real	200	local	fearful	0.11
Bailey et al. (2014)	4	30	nr	older	nr	Australia	19	nonpredictive	real	200	local	happy	0.06
Bailey et al. (2014)	4	30	nr	older	nr	Australia	20	nonpredictive	real	200	local	fearful	0.05
Bailey et al. (2014)	4	30	nr	older	nr	Australia	21	nonpredictive	real	200	local	happy	0.04
Bailey et al. (2014)	4	30	nr	older	nr	Australia	22	nonpredictive	real	200	local	fearful	0.12
Bailey et al. (2014)	4	30	nr	older	nr	Australia	23	nonpredictive	real	200	local	happy	0.05
Bailey et al. (2014)	4	30	nr	older	nr	Australia	24	nonpredictive	real	200	local	fearful	0.08
Deroche et al. (2016)	5	43	20	older	69.16	France	25	nonpredictive	generated	100	local	neutral	0.00
Deroche et al. (2016)	5	43	20	older	69.16	France	26	nonpredictive	generated	300	local	neutral	0.15
Deroche et al. (2016)	5	43	20	older	69.16	France	27	nonpredictive	generated	600	local	neutral	0.43
Deroche et al. (2016)	5	43	20	older	69.16	France	28	nonpredictive	generated	1000	local	neutral	0.14
Deroche et al. (2016)	6	43	22	young	24.05	France	29	nonpredictive	generated	100	local	neutral	0.28
Deroche et al. (2016)	6	43	22	young	24.05	France	30	nonpredictive	generated	300	local	neutral	0.36
Deroche et al. (2016)	6	43	22	young	24.05	France	31	nonpredictive	generated	600	local	neutral	0.18
Deroche et al. (2016)	6	43	22	young	24.05	France	32	nonpredictive	generated	1000	local	neutral	0.00

Gayzur et al. (2014)	7	40	24	young	20.3	United States	33	nonpredictive	schematic	100	local	neutral	0.29
Gayzur et al. (2014)	7	40	24	young	20.3	United States	34	nonpredictive	schematic	300	local	neutral	0.12
Gayzur et al. (2014)	7	40	24	young	20.3	United States	35	nonpredictive	schematic	600	local	neutral	0.15
Gayzur et al. (2014)	7	40	24	young	20.3	United States	36	nonpredictive	schematic	1000	local	neutral	0.11
Gayzur et al. (2014)	8	40	25	older	66.6	United States	37	nonpredictive	schematic	100	local	neutral	0.29
Gayzur et al. (2014)	8	40	25	older	66.6	United States	38	nonpredictive	schematic	300	local	neutral	0.22
Gayzur et al. (2014)	8	40	25	older	66.6	United States	39	nonpredictive	schematic	600	local	neutral	0.01
Gayzur et al. (2014)	8	40	25	older	66.6	United States	40	nonpredictive	schematic	1000	local	neutral	0.16
Gayzur et al. (2014)	9	40	25	older	78.9	United States	41	nonpredictive	schematic	100	local	neutral	0.06
Gayzur et al. (2014)	9	40	25	older	78.9	United States	42	nonpredictive	schematic	300	local	neutral	0.25
Gayzur et al. (2014)	9	40	25	older	78.9	United States	43	nonpredictive	schematic	600	local	neutral	0.07
Gayzur et al. (2014)	9	40	25	older	78.9	United States	44	nonpredictive	schematic	1000	local	neutral	0.01
Gayzur et al. (2014)	10	40	27	young	20.2	United States	45	nonpredictive	schematic	100	local	neutral	0.31
Gayzur et al. (2014)	10	40	27	young	20.2	United States	46	nonpredictive	schematic	300	local	neutral	0.23
Gayzur et al. (2014)	10	40	27	young	20.2	United States	47	nonpredictive	schematic	600	local	neutral	0.05
Gayzur et al. (2014)	10	40	27	young	20.2	United States	48	nonpredictive	schematic	1000	local	neutral	0.07
Gayzur et al. (2014)	11	40	27	older	67	United States	49	nonpredictive	schematic	100	local	neutral	0.18
Gayzur et al. (2014)	11	40	27	older	67	United States	50	nonpredictive	schematic	300	local	neutral	0.15
Gayzur et al. (2014)	11	40	27	older	67	United States	51	nonpredictive	schematic	600	local	neutral	0.07

Gayzur et al. (2014)	11	40	27	older	67	United States	52	nonpredictive	schematic	1000	local	neutral	0.05
Gayzur et al. (2014)	12	40	27	older	79.6	United States	53	nonpredictive	schematic	100	local	neutral	0.16
Gayzur et al. (2014)	12	40	27	older	79.6	United States	54	nonpredictive	schematic	300	local	neutral	0.19
Gayzur et al. (2014)	12	40	27	older	79.6	United States	55	nonpredictive	schematic	600	local	neutral	0.03
Gayzur et al. (2014)	12	40	27	older	79.6	United States	56	nonpredictive	schematic	1000	local	neutral	0.05
Nagy et al. (2020)	13	24	12	young	22	Hungary	57	nonpredictive	real	150	categ	Neutral or happy	-0.19
Nagy et al. (2020)	14	20	nr	older	nr	Hungary	58	nonpredictive	real	150	categ	Neutral or happy	-0.14
Slessor et al. (2010)	15	30	23	young	20.09	United Kingdom	59	nonpredictive	real	500	local	neutral	0.28
Slessor et al. (2010)	15	30	23	young	20.09	United Kingdom	60	nonpredictive	real	500	local	neutral	0.17
Slessor et al. (2010)	16	29	21	older	73.59	United Kingdom	61	nonpredictive	real	500	local	neutral	0.06
Slessor et al. (2010)	16	29	21	older	73.59	United Kingdom	62	nonpredictive	real	500	local	neutral	0.08
Slessor et al. (2008)	17	45	36	young	20.02	United Kingdom	63	predictive	real	180	local	happy	0.69
Slessor et al. (2008)	17	45	36	young	20.02	United Kingdom	64	predictive	real	180	local	sad	0.71
Slessor et al. (2008)	17	45	36	young	20.02	United Kingdom	65	predictive	real	180	local	fearful	0.67
Slessor et al. (2008)	17	45	36	young	20.02	United Kingdom	66	predictive	real	180	local	angry	0.82
Slessor et al. (2008)	17	45	36	young	20.02	United Kingdom	67	predictive	real	180	local	neutral	0.82
Slessor et al. (2008)	18	36	27	older	72.11	United Kingdom	68	predictive	real	180	local	happy	0.09
Slessor et al. (2008)	18	36	27	older	72.11	United Kingdom	69	predictive	real	180	local	sad	0.09
Slessor et al. (2008)	18	36	27	older	72.11	United Kingdom	70	predictive	real	180	local	fearful	0.17

Slessor et al. (2008)	18	36	27	older	72.11	United Kingdom	71	predictive	real	180	local	angry	0.16
Slessor et al. (2008)	18	36	27	older	72.11	United Kingdom	72	predictive	real	180	local	neutral	0.19
Slessor et al. (2016)	19	41	37	young	20.67	United Kingdom	73	predictive	real	220	local	neutral	0.64
Slessor et al. (2016)	19	41	37	young	20.67	United Kingdom	74	nonpredictive	real	220	local	neutral	0.44
Slessor et al. (2016)	20	34	28	older	72.71	United Kingdom	75	predictive	real	220	local	neutral	0.09
Slessor et al. (2016)	20	34	28	older	72.71	United Kingdom	76	nonpredictive	real	220	local	neutral	0.08
Slessor et al. (2016)	21	46	36	young	21.02	United Kingdom	77	predictive	real	220	local	neutral	0.97
Slessor et al. (2016)	22	44	37	older	72.64	United Kingdom	78	predictive	real	220	local	neutral	0.07
Dalmaso et al. (2015)	23	26	21	young	19.27	Italy	79	nonpredictive	generated	500	detect	neutral	0.13
Dalmaso et al. (2015)	24	9	3	older	63.11	Italy	80	nonpredictive	generated	500	detect	neutral	0.13
Insch (2013)	25	40	29	young	20.9	United Kingdom	81	predictive	real	200	local	neutral	0.51
Insch (2013)	26	45	33	older	72.8	United Kingdom	82	predictive	real	200	local	neutral	0.09

[1]Post data reduction only. Where the post data reduction values were not available, not reported is specified. [2]task types are localization (local), categorization (categ) and detection (det).

Supplement 4. *P*-Curve Disclosure Table

p-Curve Disclosure Table for the selection rule "Extract the p-value associated with the first test of age-differences in gaze-cueing effects with a significant (< .05) p-value"

**Citation**	**Stated Hypothesis (SH)[1]**	**Study Design**	**Statistical Result Testing SH**	**Statistical Result of Interest**	**Recomputed Precise *p*-value Based on Reported Test Statistics**	***p*-value for Inclusion in *p*-Curve**
Bailey et al. (2014) Study 1		2 (age group: young, old) X 2 (cue validity: valid, invalid) x 2 (cue presentation: subliminal, supraliminal) x 2 (face age: old, young)		"There was a congruity x participant age group interaction, $F(1, 60) = 5.41$, $p = .023$, $\eta p^2 = .08$"	*p*-value associated with $F = 5.41$ for the $F(1, 60)$ distribution.	0.023
Bailey et al. (2014) Study 2		2 (age group: young, old) X 2 (cue validity: valid, invalid) x 2 (cue presentation: subliminal, supraliminal) x 2 (face age: old, young) x 2 (face emotion: happy, fearful)		"There were, however, participant age group x congruity, $F(1, 59) = 6.58$, $p = .013$, $\eta p^2 = .10$"	*p*-value associated with $F = 6.58$ for the $F(1, 59)$ distribution.	0.013
Deroche et al. (2016)		2 (age group: young, old) x 2 (cue validity: valid, invalid) x 4 (SOAms: 100, 300, 600, 1000)		No significant difference in gaze-cueing between older and younger adults was detected overall or at any of the four SOAs.	na	
Gayzur et al. (2014) Experiment 1	"We expected that validity effects would be at least as great for older adults as for young adults".	3 (age group: young, young-old, old-old) x 2 (cue validity: valid, invalid) x 4 (SOAms: 100, 300, 600, 1000)	Age group x cue validity interaction	"Age differences in validity effects were observed at the 100-ms SOA, $F(2, 117) = 4.21$, $p = .017$. SNK analyses showed that	*p*-value associated with $F = 4.21$ for the $F(2, 117)$ distribution.	0.017

				at 100 ms, the young and young-old adults had greater validity effects than did the old-old adults.”		
Gayzur et al. (2014) Experiment 2		3 (age group: young, young-old, old-old) x 2 (cue validity: valid, invalid) x 4 (SOAms: 100, 300, 600, 1000)		“Significant age differences in the validity effect difference scores (invalid RT minus valid RT) were not found at any of the SOAs, all Fs < 1.”	na	
Nagy et al. (2020)	“We assumed that older adults, as distinct from young adults, would not be able to ignore the effect of the task-irrelevant cues…”	2 (age group: young, old) x 2 (cue validity: valid, invalid) x 2 (spatial simon condition: congruent, incongruent) x 2 (cue: face, patch)	Age group x cue validity interaction	Not assessed	na	
Slessor et al. (2010)		2 (age group: young, old) x 2 (cue validity: valid, invalid) x 2 (cue face age: young, old)		“There was also a significant Age of Participant × Cue Congruity interaction, $F(1, 56) = 7.891$, $p < .01$, $\eta_p^2 = .12$.”	*p*-value associated with $F = 7.89$ for the $F(1, 56)$ distribution.	0.007
Slessor et al. (2008) Study 2		2 (age group: young, old) x 2 (cue validity: valid, invalid) x 5 (expression: neutral, happy, sad, fearful, angry)		“A significant Cue Congruity x Age interaction was found, $F(1, 80) = 29.98$, $p < .001$, $\eta^2_p = .28$.”	*p*-value associated with $F = 29.89$ for the $F(1, 80)$ distribution.	< 0.00001
Slessor et al. (2016) Study 1		2 (age group: young, old) x 2 (cue validity: valid, invalid) x 2 (predictiveness: predictive, nonpredictive)		“These findings were qualified by a Cue-target congruency × Age group interaction,	*p*-value associated with $F = 10.78$ for the $F(1, 73)$ distribution.	0.002

				F(1,73) = 10.78, p < .01, η_p^2 = .13."		
Slessor et al. (2016) Study 2		2 (age group: young, old) x 3 (cue validity: valid, invalid, no cue)		"This was qualified by a significant Cue-target congruency × Age group interaction F(2,176) = 18.69, p < .001, η_p^2 = .18."	p-value associated with F = 18.69 for the F(2, 176) distribution.	< 0.00001
Dalmaso et al. (2015)				No direct age comparisons.	na	
Insch (2013) Chapter 7	"It is hypothesised that older adults in the current research would also follow gaze cues but to a lesser extent than the younger adults as indexed by a significantly smaller gaze congruity effect than the younger group."	2 (age group: young, old) x 2 (cue validity: valid, invalid)	Age group x cue validity interaction	"A gaze direction x age category interaction was revealed, F(1, 83) = 19.25, p < 0.001, ηp^2 = .18."	p-value associated with F = 19.25 for the F(1, 83) distribution.	0.00003

[1]If there is not SH associated with the first test of age-differences in gaze-cueing effects with a significant (< .05) p-value, cells in this table related to SH are left blank.
